# The Basophil Activation Test in the Diagnosis of Cow’s Milk Allergy in Children: A Narrative Review

**DOI:** 10.3390/nu18142217

**Published:** 2026-07-08

**Authors:** Aylin Özen, Leontien Depoorter, Koen Huysentruyt, Yvan Vandenplas

**Affiliations:** KidZ Health Castle, UZ Brussel, Vrije Universiteit Brussel (VUB), 1090 Brussels, Belgium; aylin.oezen@uzbrussel.be (A.Ö.); leontien.depoorter@uzbrussel.be (L.D.); koen.huysentruyt@uzbrussel.be (K.H.)

**Keywords:** basophil activation test, cow’s milk allergy, oral food challenge

## Abstract

**Background/Objectives:** Although cow’s milk allergy is the most common food allergy in infants, its diagnosis remains challenging. Conventional allergy tests, including skin-prick testing and serum-specific IgE, can support the diagnosis in cases with a clear clinical history; however, neither positive nor negative results are sufficient for a definitive diagnosis without confirmation via an oral food challenge or reintroduction. The basophil activation test has emerged as a promising adjunctive tool in the diagnosis of cow’s milk allergy. **Methods:** We searched in the electronic databases PubMed/MEDLINE and Google Scholar for English articles published up to March 2026, using combinations of the following keywords: “cow’s milk allergy,” “basophil activation test, “BAT”, “food allergy,” “oral food challenge,” “IgE-mediated,” “children,” and “pediatric.” **Results:** While its widespread use is currently limited by the lack of standardized protocols, the need for fresh blood samples, and restricted availability outside specialized centers, the basophil activation test has the potential to improve diagnostic accuracy and support clinical decision-making in IgE-mediated cow’s milk allergy. Importantly, basophil activation test may also reduce the number of oral food challenges by providing a safe, functional assessment of allergen reactivity, thereby minimizing patient risk and the burden of invasive testing. **Conclusions:** Further studies and validation are needed before the basophil activation test can be implemented as a routine diagnostic tool.

## 1. Introduction

Cow’s milk allergy (CMA) is one of the most common food allergies in infants and young children, typically manifesting as an adverse immune response to proteins present in cow’s milk [1]. In high-income countries, the prevalence of CMA is estimated to be approximately 2–3% [1]. CMA presents often within the first few months of life, usually within two months after the introduction of cow’s milk-based infant formula [1]. Symptoms range in severity.

The initial evaluation of suspected food allergy typically involves obtaining a detailed clinical history and performing a complete physical examination, eventually supplemented by skin-prick testing (SPT) and the measurement of allergen-specific immunoglobulin E (sIgE) levels [2]. While these diagnostic tools demonstrate high sensitivity in identifying sensitized individuals, their specificity is limited [2,3]. As a result, these tests cannot definitively confirm or exclude a diagnosis of food allergy. The Cow’s Milk-related Symptom Score (CoMiSS TM) was developed to increase awareness among healthcare professionals for CMA [4].

Currently, the oral food challenge (OFC) remains the gold standard for diagnosing CMA [5]. However, the OFC is associated with several disadvantages: it is time-consuming, resource-intensive and will cause symptoms if positive, carrying a risk of severe allergic reactions including anaphylaxis [5]. These limitations underscore the need for safer, more specific diagnostic tools that can reduce reliance on OFCs.

The basophil activation test (BAT) is a laboratory test that measures how strongly basophils react to certain allergens such as cow’s milk proteins [6]. The aim of this article is to provide an overview of the current knowledge on the use of the BAT in the diagnosis of IgE-mediated CMA in children.

## 2. Materials and Methods

This narrative review was conducted by searching PubMed/MEDLINE for articles published up to March 2026. Search terms included combinations of “cow’s milk allergy,” “basophil activation test, “BAT”, “food allergy,” “oral food challenge,” “IgE-mediated,” “children,” and “pediatric.” Additional references were identified through manual screening of the reference lists of relevant articles and review papers. Conference abstracts, editorials and letters were excluded. Only articles published in English were included. After the removal of duplicates and screening of titles and abstracts for relevance to the diagnostic role of BAT in pediatric CMA and the background information of CMA, a total of 47 references was retained and cited in this review.

Studies were excluded if they focused exclusively on adult populations, non-IgE-mediated allergy mechanism without relevance to BAT interpretation, or allergens other than cow’s milk without comparative data.

No formal risk-of-bias assessment or meta-analytic pooling was performed as this is a narrative review. Study selection was based on relevance to the research question as assessed by the authors. Given the narrative design, a formal PRISMA flow diagram was not produced; however, a summary table of key studies evaluating BAT diagnostic performance is provided.

## 3. Results

### 3.1. Cow’s Milk Allergy: Definition and Classification

CMA is caused by an adverse immune response to proteins present in cow’s milk, predominantly caseins (α1-, α2-, β-, and κ-casein) and whey proteins (α-lactalbumin and β-lactoglobulin) [7]. CMA can manifest through different immunological mechanisms, primarily classified into IgE-mediated, non-IgE-mediated, or a combination of both [7].

The incidence of CMA varies by region and diagnostic criteria. Data from the EuroPrevall birth cohort provide one of the most reliable population-based estimates, and confirmed CMA in only 0.54% of children using double-blind, placebo-controlled food challenge [8]. Interestingly, nearly one-quarter of the confirmed cases had negative cow’s milk-specific IgE (sIgE), suggesting a non-IgE-mediated mechanism [8]. These findings highlight both the relatively low prevalence of confirmed CMA and the substantial proportion of children who outgrow the condition during early childhood.

Understanding the different types and clinical presentations of CMA is crucial for effective management and improving the quality of life for affected children and their families.

### 3.2. Cow’s Milk Allergy: Pathophysiology

In IgE-mediated allergy, the immune system aberrantly produces during the “sensitization” phase IgE antibodies against cow’s milk proteins, which bind to the surface of mast cells and basophils [9]. Upon subsequent exposure, the “activation” phase occurs: IgE on mast cells recognizes allergenic epitopes, triggering rapid degranulation and the release of inflammatory mediators responsible for acute allergic reactions [9].

In contrast, non-IgE-mediated reactions are thought to result from different mechanisms, including Th1-driven immune activity and interactions between T cells, mast cells, and neurons that can alter intestinal smooth muscle function and motility [10,11]. These reactions typically develop more slowly, often hours to days after exposure, and predominantly affect the gastrointestinal tract [10,11]. The immune response involves local inflammation, epithelial damage, and the recruitment of immune cells, leading to chronic or delayed gastrointestinal symptoms [10,11].

Understanding these divergent mechanisms is crucial for the interpretation of diagnostic tools such as the BAT, which is primarily informative in IgE-mediated reactions but may provide additional insights in complex or mixed presentations.

### 3.3. Cow’s Milk Allergy: Clinical Presentation

The clinical presentation of CMA encompasses a wide spectrum of symptoms affecting multiple organ systems [12]. Gastrointestinal manifestations include diarrhea and constipation, vomiting, and colic; respiratory symptoms may present as wheezing or coughing; and cutaneous signs commonly include eczema and urticaria [7,12]. The symptom profile varies with age: infants more frequently exhibit gastrointestinal involvement, whereas older children are more likely to present with respiratory or dermatologic manifestations [11,13,14]. The heterogeneity of CMA presentations, involving various tissues and organ systems, complicates clinical identification and may contribute to diagnostic uncertainty or delayed diagnosis [11].

### 3.4. Cow’s Milk Allergy: Diagnosis

The diagnosis of CMA is primarily based on a detailed assessment of clinical symptoms in conjunction with allergy testing [11]. The 2024 ESPGHAN Position Paper discusses various tools for the diagnosis of CMA. A diagnostic elimination diet for 2 to 4 weeks, followed by a controlled oral food challenge, is recommended as the cornerstone of diagnostic confirmation, as re-exposure under supervised conditions remains the gold standard for establishing cow’s milk allergy [11].

SPT and sIgE indicate sensitization rather than clinical allergy and results should always be interpreted alongside the clinical history and, in most cases, confirmed by an elimination diet followed by a supervised oral food challenge [13]. Component-resolved diagnostics (CRD) offers a further refinement by measuring specific IgE against cow’s milk protein components [15,16]. It may help identify sensitization and predict the risk of severe reactions. However, evidence that CRD offers superior diagnostic accuracy over conventional allergy tests remains inconclusive and it cannot replace OFC [17].

While DBPCFC remains the gold standard, an open OFC is generally sufficient for confirming CMA in routine clinical practice, especially during the first year of life [13]. Despite its diagnostic value, OFC is resource-intensive, time-consuming and carries an inherent risk of allergic reactions, including anaphylaxis [18]. Across studies, allergic reaction rates during OFC range from approximately 14% to 40% and anaphylaxis occurs in 1–8% of challenges depending on patient population, the allergen tested and the clinical setting [19,20,21]. These limitations underscore the need for a non-invasive biomarker that could reduce the number of OFCs required and support decision-making [18]. More recently, the BAT has emerged as a promising diagnostic approach, offering additional insight into IgE-mediated mechanisms.

### 3.5. Basophil Activation Test

Basophils are round cells measuring approximately 5–10 μm in diameter, containing multilobed nuclei with densely packed chromatin [22].

Their characteristic appearance facilitates differentiation from other circulating leukocyte populations [23]. Histamine, present at about 1–2 pg per cell, represents the major constituent of these granules and plays a central role in the basophil’s contribution to allergic inflammation [24]. Upon activation, basophils release granule mediators into the surrounding environment through exocytosis involving multiple pores formed in the plasma membrane [24].

Classically, basophil activation and subsequent degranulation occur when high-affinity IgE receptors (FcεRI) are cross-linked by IgE antibodies bound to their specific antigen and upregulate surface activation markers including CD45, CD11b, CD11c, CD62L, CD203c, and CD63 [25]. However, basophils may also be stimulated to release mediators through IgE-independent pathways [26].

The BAT is a flow-cytometry-based assay that identifies allergen reactivity by assessing whether a suspected allergen can cross-link surface-bound IgE on peripheral blood basophils and trigger degranulation [27,28]. Introduced in 1991 as a diagnostic tool, the BAT gained momentum following the discovery of CD63 as a reliable activation marker [27,28]. CD63, normally associated with granule membranes, becomes expressed on the cell surface during degranulation and histamine release. BAT protocols employing CD63 detection typically using dual staining with anti-CD63 and anti-IgE monoclonal antibodies have demonstrated strong diagnostic accuracy for IgE-mediated allergic conditions [27,28].

Commercially available systems include Flow-CAST (Bühlmann Laboratories), BASOTEST (Becton Dickinson) and the allergenicity test (Beckman Coulter) [18,29], with each differing in their choice of basophil identification and activation markers [18,29]. Flow-CAST, the most widely used of these systems, has been released in three successive versions by using different combinations of basophil activation and identification markers. It has undergone analytical validation studies aimed at achieving compliance with the EU in In Vitro Diagnostic Regulation requirements, representing an important step towards broader regulatory recognition [18,29]. The allergenicity test identifies basophils by CRTH2 expression and measures activation through CD203c upregulation. BASOTEST uses CD63 as the activation marker with a CD123/HLA-DR gating strategy. These differences in both basophil identification and activation marker are not trivial, as they can influence assay sensitivity, specificity and inter-laboratory comparability [18,29,30].

BAT is widely used as a diagnostic tool in various food allergies. Several studies have compared the performance of the BAT to other diagnostic methods to evaluate its diagnostic accuracy and reliability (Table 1).

In the study by Ciepiela et al., the BAT was compared to sIgE for diagnosing IgE-mediated CMA in children [31]. Flow cytometry was used to analyze allergen-induced CD203c upregulation [31]. The sensitivity of this method was found to be comparable to that of traditional diagnostic techniques, while its specificity exceeded that of serum IgE testing [31]. Consequently, the BAT represents a highly effective tool for confirming CMA in pediatric patients [31].

The diagnostic performance of BAT in CMA was also compared to traditional methods, using the OFC as the reference standard in a study by Bartha et al., which focused on the ability of BAT to predict tolerance to baked milk and fresh milk in children in a cohort of 150 children [32]. BAT demonstrated the highest diagnostic accuracy for both baked milk and fresh milk allergy [32]. Using optimal cut-off values, BAT minimized the need for OFCs compared to the other tests while maintaining very high diagnostic precision; in children under 2 years of age, BAT drastically reduced the number of required OFCs and achieved 100% diagnostic accuracy for BM in this single cohort, a striking finding that requires replication in independent, larger samples before it can inform clinical practice [32]. Additionally, further analyses indicated that BAT was the only biomarker capable of distinguishing both the severity of allergic reactions and the reaction threshold during OFCs, suggesting that BAT may have the potential for enhanced risk stratification in patients with IgE-mediated CMA [32].

Beyond CMA, BAT has also been evaluated in other IgE-mediated food allergies, providing a broader context for its diagnostic potential. Santos et al. reported a sensitivity of 98% and a specificity of 96% for the BAT in diagnosing peanut allergy in peanut-sensitized children [33]. Similarly, Berghea et al. demonstrated a sensitivity of 85.0% and a specificity of 77.0% when applying the test to wheat allergy [34]. While these findings support the general principle that BAT can discriminate allergy from sensitization across multiple allergens, they should not be interpreted as direct evidence for its performance in CMA. Allergen-specific differences mean that diagnostic accuracy established for peanut or wheat cannot be assumed to apply to cow’s milk.

The BAT is not only a diagnostic tool but can also be used to assess the severity of allergic reactions [33,35,36]. According to the study by Rubio et al., a strong correlation was observed between the severity of clinical allergic reactions and the extent of basophil activation, as reflected by each patient’s individual basophil reactivity [36]. Another study, performed by S. Ford et al., also suggests that the BAT provides insights into the severity of clinical reactivity and the degree of allergen tolerance [35]. However, as discussed further below, these findings have not been consistently replicated and should be regarded as hypothesis-generating rather than practice-defining.

Several studies have also highlighted the added value of BAT in guiding OFCs [33,37]. In the study by Ruinemans–Koerts et al., 80% of children showed complete concordance between the results of the DBPCFC and the BAT [37]. The BAT provides a safe in vitro alternative to the DBPCFC that can accurately identify IgE-mediated CMA without exposing children to an oral allergen challenge, though this conclusion is based on a single-center study and requires external validation before it can be generalized [37]. BAT can substantially reduce the need for DBPCFC, reserving oral challenges only for selected cases where immunological mechanisms remain unclear [33,37]. Given these findings, the BAT appears not only valuable for determining the optimal timing of OFC but also potentially useful for monitoring the progression of desensitization and the development of tolerance in children undergoing therapeutic interventions [32,33,37] (Table 1).

Taken together, these studies suggest that the BAT may offer diagnostic accuracy comparable to and, in several populations, exceeding that of SPT and sIgE, while simultaneously providing functional information on the reaction severity and threshold that the conventional test cannot deliver. These conclusions should be interpreted with caution, given that the most evidence originates from single-center studies with limited external validation, while standardized cut-off values are lacking and current international guidelines continue to classify OFC as the diagnostic gold standard. BAT should therefore be regarded as a promising adjunctive tool rather than a replacement for OFC in routine clinical practice.

#### The Diagnostic Value of BAT Baked Versus Fresh Milk

The diagnostic value of the BAT, however, is not uniform across all clinical presentations of IgE-mediated CMA and its interpretation differs depending on whether baked or fresh milk allergy is being assessed. This distinction has both immunological and clinical dimensions. Ford et al. demonstrated across 132 children that basophil reactivity, casein-specific IgE, and milk SPT wheal size all differed significantly across five clinical tolerance groups, and were markedly higher in baked milk-reactive compared to baked milk-tolerant children [35]. In the BAT2 study, BAT was the only biomarker able to predict both the severity and the reaction threshold for baked and fresh milk challenges, but the performance profiles diverged: for baked milk, BAT achieved 71% sensitivity and 100% specificity in identifying severe reactors, reflecting the narrower, more extreme phenotype of children who react to heat-denatured protein [32]. For fresh milk, the picture was different. BAT reached 96% sensitivity for identifying low-threshold reactors, but specificity fell to only 41%, because the much broader range of sensitization among fresh milk-reactive children makes it harder to discriminate [32].

This performance asymmetry has a practical implication: a positive BAT result carries a different predictive weight depending on which allergen form is being challenged. In the context of baked milk, a positive BAT result identifies a small, high-risk subgroup in whom a reaction during OFC is both probable and likely to be severe [32]. For fresh milk, BAT’s high sensitivity makes it a strong rule-out tool, but its modest specificity means a positive result alone does not reliably define who will react clinically [32]. These differences underscore that BAT cut-offs and clinical interpretation cannot be applied uniformly across challenge types, and that future standardization efforts should define type-specific thresholds separately.

**Table 1:** Summary of key findings regarding basophil activation test performance.

Study	Design	N°	Population	BAT Marker	Allergen Stimulus	Reference Standard	Sensitivity	Specificity	Key Finding
Ciepiela et al. (2010) [31]	Prospective	24 (9 CMA, 15 control)	suspected IgE-CMA	CD203c	CM extract (1:10 and 1:500 dilutions)	sIgE/clinical diagnosis	Comparable to sIgE	Higher than sIgE	CD203c upregulation correctly identified 7/9 allergic children; no activation above cut-off in controls
Ruinemans-Koerts et al. (2019) [37]	Prospective	86 infants/young children	suspected (persistent) CMA	CD63	CM extract and purified allergens	DBPCFC	100% in IgE-sensitized children	100% in IgE-sensitized children	BAT correctly identified all allergic children; reduces need for OFC in IgE-mediated CMA
Ford et al. (2013) [35]	Cross-sectional	132	IgE-CMA, classified into 5 tolerance groups	CD63	Casein, whole milk powder	Clinical tolerance groups	NA	NA	Significant graded relationship between basophil reactivity and the degree of milk tolerance
Bartha et al. (2025) [32]	Prospective cohort	150	CMA undergoing OFC	CD63/CD203c	Baked milk, fresh milk	OFC	71% (BM severe)/96% (FM low threshold)	100% (for BM)/41% (for FM)	BAT is the most accurate predictor of OFC outcome; it is the only biomarker to predict severity and reaction threshold
Santos et al. (2014) * [33]	Prospective	104 children + 65 validation cohort	43 peanut-allergic, 36 sensitized-tolerant, 25 non-sensitized	CD63 and CD203c	Peanut extract (100 ng/mL and serial dilutions)	DBPCFC/ convincing history/peanut-sIgE ≥ 15 kU/L	CD63 97.6% CD203c 95.2%	CD6396% CD203c 96%	Reduced need for OFC by two-thirds, validate in independent cohort
Berghea et al. (2025) * [34]	Narrative review	—	Children/adults with wheat allergy	CD63/CD203c	Wheat extract	OFC	85%	77%	BAT applicable to wheat allergy diagnosis

Legend: * Studies involving food allergies other than CMA, included for illustrative purposes. Findings should not be extrapolated directly to CMA. N/A = not applicable; BM = baked milk; FM = fresh milk; N°: number of patients; BAT: basophil activation test; DBPCFC: double-blind placebo-controlled food challenge; OFC: oral food challenge; CM(A): cow’s milk (allergy).

### 3.6. Limitations of the BAT

The substantial methodological heterogeneity in the published literature is a critical challenge in interpreting the diagnostic performance of BAT across studies. Reported sensitivity and specificity values vary considerably and this variability cannot be attributed solely to differences in patient populations. Key sources of heterogeneity include the choice of activation marker (CD63 versus CD203c), allergen preparation (whole-extract stimulation versus individual molecular components) stimulation protocol (single-concentration assays versus serial log-dilution titration) and positivity cut-off values, which are currently not standardized and are often derived within individual studies, limiting cross-study comparability.

The BAT is not yet universally used for diagnosing food allergies, despite its high diagnostic potential, due to several limitations and challenges. There is a lack of validation and standardization, which prevents the universal application of the test across different laboratories and protocols [30]. A detailed protocol is needed to determine which allergens should be used and how relevant they are for different patient groups. It is also essential to identify which activation markers are most effective in measuring responses across various patient populations [30]. According to a study, 5–10% of patients have non-responsive basophils, which do not upregulate CD203c or CD63 when stimulated by IgE-mediated allergens, further limiting the implementation of BATs [38]. Furthermore, basophil responses can be influenced by the patient’s medication use and clinical condition, while variability in allergen extracts may reduce the reproducibility of the results [38].

The choice of stimulant, whole-allergen extract versus isolated molecular components (recombinant or purified), has important consequences for BAT performance, particularly in CMA. Whole-protein extracts contain the full complement of allergen molecules and their native conformational structures, and as such tend to elicit stronger basophil responses [39].

For cow’s milk specifically, extract-based stimulation was confirmed as superior: Chhing et al. showed that even in concordant cases, component-stimulated basophils displayed on average 6% lower activation rates than their extract-stimulated counterparts and that discordance was more pronounced in patients with lower total IgE [39]. This has implications for standardization: laboratories using component-based protocols for cow’s milk BAT should be aware of the elevated risk of false-negative results, particularly in patients with lower total IgE or undergoing immunotherapy.

Basophils can be stimulated with single-allergen concentration or with a serial log-dilution series [40,41,42]. The single-concentration approach is operationally simpler, faster, and less costly: one concentration of allergen is applied and the percentage of CD63- or CD203c-positive basophils is read as the primary outcome [42]. This reflects how strongly basophils respond at a given dose. Its main limitation is that it is sensitive to the chosen concentration: too low and even truly allergic patients may not show activation; too high and the dose–response plateau may obscure differences between patients with varying degrees of sensitization [40,41,42]. A serial log titration approach addresses this by generating the full dose–response curve, that reflects both reactivity and sensitivity and may improve discrimination between tolerant and reactive patients, as well as between mild and severe reactors [40,41,42]. Increased cost, complexity and processing time limit feasibility in routine clinical settings [40,42].

A further practical limitation relates to the stability of basophils ex vivo. Traditionally, BAT required analysis within 4–6 h of blood collection, which restricts the test to centers with on-site flow cytometry capacity [43,44,45]. With recent developments, CD203c upregulation remains stable in EDTA or heparin-anticoagulated blood stored at 4 °C for up to 24 h [43,44,45]. More recently studies showed that processed samples can be stored at room temperature or 2–8 °C for up to 7 days before analysis, and cryopreservation approaches using glycerol-assisted protocols at −20 °C or glycerol-free storage at −80 °C have shown feasibility for long-term storage of up to 6–12 months [43,44,45]. These advances are relevant for multicenter studies.

The practical implementation of BAT in routine clinical settings faces several additional barriers. Inter-laboratory variability remains a significant concern: even when the same commercial platform is used, differences in operator technique, flow cytometer calibration and gating strategies can produce divergent results, underscoring the urgent need for external quality assurance schemes [42]. The EAACI task force consensus protocol published in 2024 represents an important step towards harmonization, but uptake across centers is not yet uniform [40]. BAT availability to specialized centers in many healthcare systems is restricted by equipment requirements including access to a calibrated flow cytometer and trained personnel capable of processing samples within defined time windows [42].

The reimbursement and formal regulatory recognition of the BAT remain limited in many healthcare systems [46]. In several countries, the test does not yet meet the criteria required for inclusion under standard health insurance coverage, reflecting the broader need for prospective clinical validation and harmonized protocols. The cost of the test varies depending on the center and the type of allergy [46]. Formal health-economic evaluations are warranted: while BAT has the potential to reduce the number of resource-intensive OFCs performed, the costs of equipment, personnel training and quality assurance must be weighed against these savings and no published cost-effectiveness analysis specifically addressing BAT in pediatric CMA was identified in this review.

### 3.7. The Basophil Activation Test and Oral Immune Therapy

While elimination diets remain the cornerstone of management, they do not actively alter the natural course of the allergy. This has led to growing interest in approaches that aim to accelerate tolerance acquisition, most notably oral immunotherapy (OIT) [47,48].

OIT may be considered for children with confirmed IgE-mediated CMA, particularly when dietary avoidance proves insufficient, negatively affects daily functioning, or significantly diminishes quality of life [47,49]. According to the EAACI Guidelines, OIT can be offered to children from approximately 4 to 5 years of age with persistent CMA, with the intention of raising the reaction threshold and reducing the likelihood of accidental reactions [49]. This recommended age range reflects the observation that most children who naturally outgrow CMA typically achieve tolerance before entering school [49].

Several studies have explored the use of BAT as a biomarker for monitoring immunological changes during OIT. Broadly, a reduction in basophil activation has been observed over the course of allergen-specific immunotherapy in patients with food allergies including cow’s milk, peanut and egg, suggesting that BAT may reflect the immunological modulation induced by OIT and could, in principle, reduce the number of OFCs required to assess treatment response [50]. The study by Giulia et al. provides a synthesis of BAT applications in pediatric allergic disease, including its potential monitoring role during OIT [50].

However, the current evidence base in this area remains limited and should be interpreted with caution. Most studies are small in sample size, heterogeneous in their OIT protocols and vary in BAT markers and the cut-off values employed. Longitudinal data tracking and the correlation of these changes with clinical outcomes such as sustained unresponsiveness are sparse. It is therefore premature to recommend BAT as a validated monitoring tool in routine OIT management. Its role in this context remains investigational and robust prospective multicenter trials with standardized protocols are necessary before definitive conclusions can be drawn.

## 4. Discussion

The evidence reviewed in this article pertains exclusively to IgE-mediated CMA. The BAT is mechanistically grounded in FcεRI cross-linking and basophil degranulation and its diagnostic utility is therefore primarily established in IgE-mediated forms of CMA. Its role in non-IgE-mediated presentations remains limited and poorly defined; clinicians should exercise care not to apply BAT results in patients whose presentation suggests a non-IgE-mediated mechanism.

The available literature indicates that the BAT demonstrates particularly high specificity and may therefore more effectively discriminate between mere sensitization and clinically relevant IgE-mediated CMA compared with conventional IgE-based diagnostics [31,33]. This distinction is of considerable clinical importance, especially in patients presenting with discordant findings between clinical history and sIgE levels. In such cases, BAT has been proposed as a valuable adjunctive diagnostic tool, particularly when the indication for an OFC is uncertain and the risk–benefit balance requires careful consideration.

Several studies have reported an association between increased basophil activation and greater clinical reaction severity [32,33]. However, these findings have not been consistently replicated across independent cohorts and substantial variability exists in how reaction severity is defined and graded across studies. The activation markers, allergen preparations and cut-off values used also differ considerably, further complicating interpretation. BAT should therefore currently be regarded as a promising investigational biomarker for severity assessment rather than an established clinical predictor. Prospective studies with prespecified, harmonized severity grading and standardized BAT protocols are needed before this application can be validated [30].

The reported sensitivities and specificities vary substantially across studies. Understanding the reasons for this variability is essential before BAT can be incorporated into a diagnostic algorithm. Several interacting sources of heterogeneity explain this variability. First, the choice of activation marker is not trivial. CD63 and CD203c are not interchangeable and cut-off values derived in one marker system cannot be assumed to apply to the other. Studies reporting high sensitivity with CD203c may therefore not be directly comparable to those reporting high specificity with CD63, even applied to similar patient populations. A second source of variability is allergen preparation. As mentioned above, whole-protein extracts contain the full complement of allergen molecules in their native conformational structure and tend to elicit stronger basophil responses than isolated molecular components; the use of the isolated molecular components can reduce reported sensitivity without any change in patient population. Third, stimulation design, particularly the use of single-allergen concentration versus serial-allergen dilutions, may substantially influence sensitivity and specificity. Single-concentration protocols are more susceptible during under- or overstimulation depending on the chosen allergen dose, whereas serial dilution approaches provide additional information on basophil sensitivity and reactivity that may improve diagnostic discrimination. Finally, the absence of standardized diagnostic thresholds means that cut-off values are typically from the specific study population, optimized by ROC analysis and not validated externally. A threshold that achieves 97% accuracy in one cohort may perform substantially differently in a population with different characteristics.

In the context of the OIT, dynamic changes in basophil reactivity suggest that the BAT may serve as a biomarker for immunological modulation and treatment response [50]. Nonetheless, current evidence remains limited and is derived from relatively small and heterogeneous study populations [30]. Robust longitudinal data are necessary before the BAT can be reliably implemented as a monitoring tool in clinical practice [30].

Despite its promising diagnostic performance, widespread clinical implementation of the BAT is currently hindered by the lack of international standardization [30,42]. Consensus on allergen sources and preparation, activation markers, assay protocols and diagnostic cut-off values is essential to ensure reproducibility and inter-laboratory comparability [30,42]. Beyond standardization, real-world implementation is further constrained by the requirement for specialized flow cytometry equipment and trained personnel, which limits access to tertiary centers in most healthcare systems [42]. Economic evaluations are currently lacking and are needed to determine whether BAT implementation is cost-effective compared with the current reliance on OFCs, taking into account not only direct healthcare costs but also patient burden and resource utilization [42,46].

Future research addressing diagnostic accuracy, prognostic value, therapeutic monitoring, and health-economic impact may further support the integration of the BAT into personalized allergy management strategies.

## 5. Conclusions

In conclusion, the BAT represents a promising adjunctive tool in the diagnosis of IgE- mediated CMA. Its high specificity may enable more accurate discrimination between sensitization and clinically relevant allergies compared with conventional IgE-based tests, thereby reducing unnecessary OFCs and improving risk stratification in selected patients.

However, the current evidence must be interpreted with appropriate caution. Considerable heterogeneity exists across published studies, driven by differences in allergen preparations, activation markers (CD63 versus CD203c), stimulation protocols and positivity cut-off values, which limits the direct comparability of reported diagnostic performance. Evidence extrapolated from other food allergies, such as peanut and wheat allergy, provides supportive context, but cannot be assumed to apply directly to CMA. The association between basophil activation and clinical reaction severity, while observed in several studies, has not been consistently replicated and BAT should therefore be regarded as an investigational rather than an established predictor of severity or the reaction threshold. Similarly, its potential role as a monitoring biomarker during oral immunotherapy is biologically plausible and supported by preliminary data, but remains insufficiently validated for routine clinical use. The scope of this review is restricted to IgE-mediated CMA; the utility of BAT in non-IgE-mediated presentations is not established.

Practical barriers, including inter-laboratory variability, lack of international standardization, equipment and personnel requirements and limited reimbursement and regulatory recognition in many healthcare systems further constrain widespread implementation at present.

Well-designed prospective multicenter studies with harmonized BAT protocols, prespecified severity grading and longitudinal follow-up are essential to firmly establish the position of BAT within the diagnostic algorithm of IgE-mediated CMA and to support its eventual integration into routine clinical practice and personalized allergy management.

## Data Availability

No new data were created or analyzed in this study. Data sharing is not applicable to this article.

## Abbreviations

The following abbreviations are used in this manuscript:

APC	antigen-presenting cell
BAT	basophil activation test
CI	confidence interval
CMA	cow’s milk allergy
CoMiSS	Cow’s Milk-related Symptom Score
CRD	Component-resolved diagnostics
DBPCFC	double-blind, placebo-controlled food challenge
OFC	oral food challenge
OIT	oral immunotherapy
sIgE	specific Immunoglobulin E
SPT	skin-prick test

## References

[1] Edwards C.W., Younus M.A. (2025). Cow Milk Allergy. StatPearls.

[2] Oriel R.C., Wang J. (2021). Diagnosis and Management of Food Allergy. Immunol. Allergy Clin. N. Am..

[3] Alpan O., Wasserman R.L., Kim T., Darter A., Shah A., Jones D., McNeil D., Li H., Ispas L., Rathkopf M. (2023). Towards an FDA-Cleared Basophil Activation Test. Front. Allergy.

[4] Vandenplas Y., Zhao Z.-Y., Mukherjee R., Dupont C., Eigenmann P., Kuitunen M., Ribes Koninckx C., Szajewska H., Von Berg A., Bajerová K. (2022). Assessment of the Cow’s Milk-Related Symptom Score (CoMiSS) as a Diagnostic Tool for Cow’s Milk Protein Allergy: A Prospective, Multicentre Study in China (MOSAIC Study). BMJ Open.

[5] Calvani M., Bianchi A., Reginelli C., Peresso M., Testa A. (2019). Oral Food Challenge. Medicina.

[6] Uyttebroek A.P., Sabato V., Faber M.A., Cop N., Bridts C.H., Lapeere H., De Clerck L.S., Ebo D.G. (2014). Basophil Activation Tests: Time for a Reconsideration. Expert Rev. Clin. Immunol..

[7] Hao L., Wang S., Ji W. (2025). Cow’s Milk Protein Allergy: A Comprehensive Review of Epidemiology, Pathogenesis, Clinical Manifestations, Diagnostics, and Management Strategies. Asia Pac. J. Clin. Nutr..

[8] Schoemaker A.A., Sprikkelman A.B., Grimshaw K.E., Roberts G., Grabenhenrich L., Rosenfeld L., Siegert S., Dubakiene R., Rudzeviciene O., Reche M. (2015). Incidence and Natural History of Challenge-proven Cow’s Milk Allergy in European Children—EuroPrevall Birth Cohort. Allergy.

[9] Romagnani S. (2000). The Role of Lymphocytes in Allergic Disease. J. Allergy Clin. Immunol..

[10] Lee J.H., Noh G., Noh J., Lee S., Choi W.S., Kim H.S., Lee K., Choi S., Jin H., Cho S. (2010). Clinical Characteristics of Oral Tolerance Induction of IgE-Mediated and Non-IgE-Mediated Food Allergy Using Interferon Gamma. Allergy Asthma Proc..

[11] Vandenplas Y., Broekaert I., Domellöf M., Indrio F., Lapillonne A., Pienar C., Ribes-Koninckx C., Shamir R., Szajewska H., Thapar N. (2024). An ESPGHAN Position Paper on the Diagnosis, Management, and Prevention of Cow’s Milk Allergy. J. Pediatr. Gastroenterol. Nutr..

[12] Flom J.D., Sicherer S.H. (2019). Epidemiology of Cow’s Milk Allergy. Nutrients.

[13] Giannetti A., Toschi Vespasiani G., Ricci G., Miniaci A., Di Palmo E., Pession A. (2021). Cow’s Milk Protein Allergy as a Model of Food Allergies. Nutrients.

[14] Lifschitz C., Szajewska H. (2015). Cow’s Milk Allergy: Evidence-Based Diagnosis and Management for the Practitioner. Eur. J. Pediatr..

[15] Popielarz M., Krogulska A. (2021). The Importance of Component-Resolved Diagnostics in IgE-Mediated Cow’s Milk Allergy. Allergol. Immunopathol..

[16] Bartuzi Z., Cocco R.R., Muraro A., Nowak-Węgrzyn A. (2017). Contribution of Molecular Allergen Analysis in Diagnosis of Milk Allergy. Curr. Allergy Asthma Rep..

[17] Fiocchi A., Bouygue G.R., Albarini M., Restani P. (2011). Molecular Diagnosis of Cow’s Milk Allergy. Curr. Opin. Allergy Clin. Immunol..

[18] Ferrer M., Sanz M.L., Sastre J., Bartra J., del Cuvillo A., Montoro J., Jáuregui I., Dávila I., Mullol J., Valero A. (2009). Molecular Diagnosis in Allergology: Application of the Microarray Technique. J. Investig. Allergol. Clin. Immunol..

[19] Akuete K., Guffey D., Israelsen R.B., Broyles J.M., Higgins L.J., Green T.D., Naimi D.R., MacGinnitie A.J., Vitalpur G., Minard C.G. (2017). Multicenter Prevalence of Anaphylaxis in Clinic-Based Oral Food Challenges. Ann. Allergy. Asthma. Immunol..

[20] Sengul Emeksiz Z., Ertugrul A., Ozmen S., Cavkaytar O., Ercan N., Bostancı İ.B. (2021). Is Oral Food Challenge as Safe Enough as It Seems?. J. Trop. Pediatr..

[21] Dhar A., Liao L., Liem J., Sidhu A., Kassum S. (2016). Oral Food Challenges: A Retrospective Review of A Canadian Paediatric Allergy Clinic. Paediatr. Child Health.

[22] Chirumbolo S. (2012). State-of-the-Art Review about Basophil Research in Immunology and Allergy: Is the Time Right to Treat These Cells with the Respect They Deserve?. Blood Transfus..

[23] Cromheecke J.L., Nguyen K.T., Huston D.P. (2014). Emerging Role of Human Basophil Biology in Health and Disease. Curr. Allergy Asthma Rep..

[24] Knol E.F., Olszewski M. (2011). Basophils and Mast Cells: Underdog in Immune Regulation?. Immunol. Lett..

[25] Dvorak A.M. (1994). Similarities in the Ultrastructural Morphology and Developmental and Secretory Mechanisms of Human Basophils and Eosinophils. J. Allergy Clin. Immunol..

[26] Varricchi G., Raap U., Rivellese F., Marone G., Gibbs B.F. (2018). Human Mast Cells and Basophils—How Are They Similar How Are They Different?. Immunol. Rev..

[27] Boumiza R., Debard A.-L., Monneret G. (2005). The Basophil Activation Test by Flow Cytometry: Recent Developments in Clinical Studies, Standardization and Emerging Perspectives. Clin. Mol. Allergy.

[28] Mukai K., Gaudenzio N., Gupta S., Vivanco N., Bendall S.C., Maecker H.T., Chinthrajah R.S., Tsai M., Nadeau K.C., Galli S.J. (2017). Assessing Basophil Activation by Using Flow Cytometry and Mass Cytometry in Blood Stored 24 Hours before Analysis. J. Allergy Clin. Immunol..

[29] Bunyavanich S., Akdis M., Berin M.C., Eggel A., Patil S.U., Shamji M.H., López J., Sampson H.A. (2025). Novel Biomarkers and Diagnostic Tools in Food Allergy. Allergy.

[30] Briceno Noriega D., Teodorowicz M., Savelkoul H., Ruinemans-Koerts J. (2021). The Basophil Activation Test for Clinical Management of Food Allergies: Recent Advances and Future Directions. J. Asthma Allergy.

[31] Ciepiela O., Zwiazek J., Zawadzka-Krajewska A., Kotula I., Kulus M., Demkow U. (2010). Basophil Activation Test Based on the Expression of CD203c in the Diagnostics of Cow Milk Allergy in Children. Eur. J. Med. Res..

[32] Bartha I., Boyd H., Foong R., Krawiec M., Marques-Mejias A., Marshall H.F., Radulovic S., Harrison F., Antoneria G., Jama Z. (2025). The Basophil Activation Test Is the Most Accurate Test in Predicting Allergic Reactions to Baked and Fresh Cow’s Milk During Oral Food Challenges. Allergy.

[33] Santos A.F., Douiri A., Bécares N., Wu S.-Y., Stephens A., Radulovic S., Chan S.M.H., Fox A.T., Du Toit G., Turcanu V. (2014). Basophil Activation Test Discriminates between Allergy and Tolerance in Peanut-Sensitized Children. J. Allergy Clin. Immunol..

[34] Berghea E.C., Coman-Stanemir M., Papacocea I.R. (2025). Basophil Activation Test in IgE-Mediated Wheat Allergy: Diagnostic and Clinical Applications—A Narrative Review. Diagnostics.

[35] Ford L.S., Bloom K.A., Nowak-Węgrzyn A.H., Shreffler W.G., Masilamani M., Sampson H.A. (2013). Basophil Reactivity, Wheal Size, and Immunoglobulin Levels Distinguish Degrees of Cow’s Milk Tolerance. J. Allergy Clin. Immunol..

[36] Rubio A., Vivinus-Nébot M., Bourrier T., Saggio B., Albertini M., Bernard A. (2011). Benefit of the Basophil Activation Test in Deciding When to Reintroduce Cow’s Milk in Allergic Children: Basophil Activation Test in Cow’s Milk Allergy. Allergy.

[37] Ruinemans-Koerts J., Schmidt-Hieltjes Y., Jansen A., Savelkoul H.F.J., Plaisier A., Van Setten P. (2019). The Basophil Activation Test Reduces the Need for a Food Challenge Test in Children Suspected of IgE-mediated Cow’s Milk Allergy. Clin. Exp. Allergy.

[38] Kim Y.H., Kim Y.S., Park Y., Kim S.Y., Kim K.W., Kim H.S., Sohn M.H. (2020). Investigation of Basophil Activation Test for Diagnosing Milk and Egg Allergy in Younger Children. J. Clin. Med..

[39] Chhing A., Choi S., Khelifi-Touhami D., Abbas A., Ballot E., Cottel N., Saf S., Germain G., Lambert N., Challier P. (2026). Allergen Extract Outperforms Molecular Components in Basophil Activation Test in a Pediatric Cohort. Front. Immunol..

[40] Pascal M., Edelman S.M., Nopp A., Möbs C., Geilenkeuser W.J., Knol E.F., Ebo D.G., Mertens C., Shamji M.H., Santos A.F. (2024). EAACI Task Force Report: A Consensus Protocol for the Basophil Activation Test for Collaboration and External Quality Assurance. Allergy.

[41] Bergmann M.M., Santos A.F. (2024). Basophil Activation Test in the Food Allergy Clinic: Its Current Use and Future Applications. Expert Rev. Clin. Immunol..

[42] Sonder S.U., Plassmeyer M., Schroeder N., Peyton S., Paige M., Girgis M., Safi H., Alpan O. (2025). Basophil Activation Test; User’s Manual. J. Immunol. Methods.

[43] Agyemang A., Suprun M., Suarez-Farinas M., Boina F., Arif R., Grishin A.V., Busnel J.-M., Sampson H.A. (2019). A Novel Approach to the Basophil Activation Test (BAT) for Characterizing Peanut Allergic Patients in the Clinical Setting. J. Allergy Clin. Immunol..

[44] Bourgoin P., Dupont T., Agabriel C., Carsin A., Verles A., Cabanski M., Vitaliti A., Busnel J. (2024). Possible Alternative Strategies to Implement Basophil Activation Testing in Multicentric Studies. Cytom. B Clin. Cytom..

[45] Castaing-Lasvignottes M., Bourgoin P., Carsin A., Dubus J., Busnel J. (2025). Cryopreservation Between Sample Processing and Analysis in Streamlined Basophil Activation Test. Allergy.

[46] Zorginstituut Nederland (2021). Veelbelovende Zorg—BAT Bij Kinderen Met Mogelijke IgE Gemedieerde Koemelkallergie.

[47] Mori F., Barni S., Liccioli G., Novembre E. (2019). Oral Immunotherapy (OIT): A Personalized Medicine. Medicina.

[48] Sánchez-García S., Cipriani F., Ricci G. (2015). Food Allergy in Childhood: Phenotypes, Prevention and Treatment. Pediatr. Allergy Immunol..

[49] Pajno G.B., Fernandez-Rivas M., Arasi S., Roberts G., Akdis C.A., Alvaro-Lozano M., Beyer K., Bindslev-Jensen C., Burks W., Ebisawa M. (2018). EAACI Guidelines on Allergen Immunotherapy: IgE-mediated Food Allergy. Allergy.

[50] Giulia D., Paola D.F., Armando D.L., Pasquale S., Domenico D.B., Francesca D., Sabrina D.P., Francesco C., Paola L., Marina A. (2024). Applications of Basophil Activation Test in Paediatric Allergic Diseases. World Allergy Organ. J..

